# Effect of diabetic kidney disease on therapeutic strategies for coronary artery disease: ten year follow-up

**DOI:** 10.18632/aging.203476

**Published:** 2021-08-25

**Authors:** Daniel Valente Batista, Whady Hueb, Eduardo Gomes Lima, Paulo Cury Rezende, Cibele Larrosa Garzillo, Rosa Maria Rahmi Garcia, Jaime Paula Pessoa Linhares Filho, Eduardo Bello Martins, Carlos Vicente Serrano Junior, Jose Antonio Franchini Ramires, Roberto Kalil Filho

**Affiliations:** 1 Instituto do Coracao (InCor), Hospital das Clinicas HCFMUSP, Faculdade de Medicina, Universidade de Sao Paulo, SP, BR.

**Keywords:** coronary artery disease, chronic renal failure, type 2 diabetes, cardiac surgery

## Abstract

Background: The best treatment for coronary artery disease (CAD) in patients with type 2 diabetes (DM2) and chronic kidney disease is unknown.

Methods: This retrospective study included MASS registry patients with DM2 and multivessel CAD, stratified by kidney function. Primary endpoint was combined of mortality, myocardial infarction, or additional revascularization.

Results: Median follow-up was 9.5 years. Primary endpoint occurrences among strata 1 and 2 were 53.4% and 40.7%, respectively (P=.020). Mortality rates were 37.4% and 24.6% in strata 1 and 2, respectively (P<.001). We observed a lower rate of major adverse cardiovascular events (MACE) (P=.027 for stratum 1 and P<.001 for stratum 2) and additional revascularization (P=.001 for stratum 1 and P<.001 for stratum 2) for those in the surgical group. In a multivariate analysis, eGFR was an independent predictor of MACE (P=.034) and mortality (P=.020).

Conclusions: Among subjects with DM2 and CAD the presence of lower eGFR rate was associated with higher rates of MACE and mortality, irrespective of treatment choice. CABG was associated with lower rates of MACE in both renal function strata. eGFR was an independent predictor of MACE and mortality in a 10-year follow-up.

## INTRODUCTION

Type 2 diabetes mellitus is a highly prevalent disease and is associated with macro and microvascular involvement [1]. The microvascular impairment could occur in the kidney glomerulus, resulting in diabetic kidney nephropathy [2].

The association between type 2 diabetes and kidney disease is widely recognized and increases through age [3]. Data from the NANHES study [4] show that almost 25% of diabetic patients have some degree of renal dysfunction, which is a rate almost 5 times higher than that of the non-diabetic population. In addition, even after adjusting for demographic variables, 24% of the population with chronic kidney disease (CKD) had type 2 diabetes as the main etiology. When analyzing data from diabetic patients from an Italian Cohort, the prevalence of eGFR less than 60 mL/min/1.73m^2^ among those older than 75 years was almost 45% [3].

This association plays a role in the promotion and progression of coronary artery disease (CAD), both by factors intrinsic to diabetes and by conditions related to kidney disease [5]. In addition, both clinical and interventional treatment, whether percutaneous coronary intervention (PCI) or coronary artery bypass graft (CABG), are impaired in this population [6]. In a subanalysis of the BARI trial, patients with CAD associated with type 2 diabetes and CKD had a 7-year survival of only 33% [7].

Despite its clinical relevance, available data regarding the subject are sparse. Patients with any degree of kidney disease were excluded from almost 80% of studies on CAD [8] In addition, most reports have a short follow-up, with most having less than 3 years [9].

In this context, a longer follow-up time would allow, on the one hand, to know in detail the possible long-term impacts of a given treatment strategy in relation to its benefits, and on the other hand, a better understanding of the occurrence of its potential harm [10]. It is noteworthy that, after 5 years of follow-up, atherosclerotic disease may progress in native territories, in beds submitted to percutaneous treatment, through neoatherosclerosis, as well as in grafts of patients undergoing surgical treatment.

Thus, the objective of this study was to compare very long-term outcomes among diabetic patients with stable CAD, stratified according to their renal function, who were enrolled in the MASS registry and underwent CABG, PCI, or medical therapy (MT).

## MATERIALS AND METHODS

### Study design

**Figure 1:**
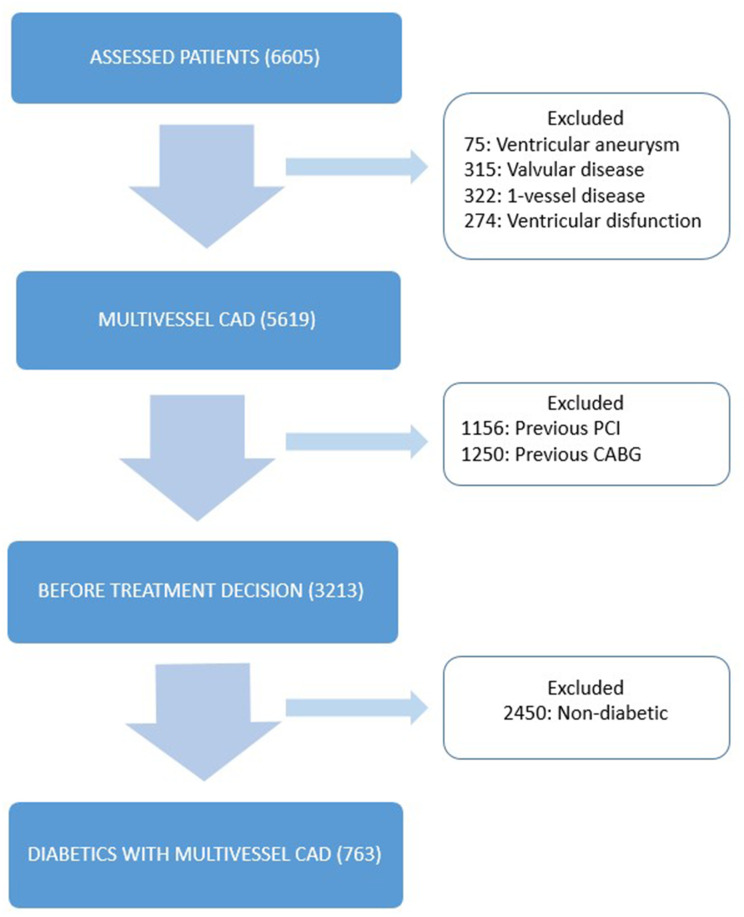
**Study design.** Flow-chart showing selection of patients. CABG, coronary artery bypass surgery; CAD: coronary artery disease; PCI: percutaneous coronary intervention.

This was a single-center, prospective, registry-based study that enrolled patients from the MASS Group Database at the Heart Institute of the University of São Paulo. The MASS Registry comprises patients with CAD assessed by our study group after the patients underwent angiography (MASS II registration number ISRCTN66068876). For this analysis, patients with multivessel CAD, type 2 diabetes, and preserved ventricular function were allocated to one of the treatment group options: CABG, PCI, or MT (Figure 1). Data were analyzed according to estimated glomerular filtration rate (eGFR) using the Chronic Kidney Disease Epidemiology Collaboration (CKD-EPI) equation formula, resulting in 2 strata: eGFR ≥ 60 mL/min/1.73m^2^ and eGFR < 60 mL/min/1.73m^2^. Type 2 diabetes was defined according to definitions proposed by the American Diabetes Association [11]. Multivessel CAD was defined as those with > 70% obstruction on angiography in at least 2 major coronary arteries or their major branches. An informed consent was obtained from all participants.

Exclusion criteria included unstable angina or acute MI, ventricular aneurysm requiring surgical repair, left ventricular ejection fraction of less than 40%, previous PCI or CABG, single-vessel disease, or any previous cardiac surgery, eGFR < 30 mL/min/1.73m^2^, patients already on dialysis, or those after kidney transplantation.

We calculated the eGFR based on the CKD-EPI equation, as suggested by the National Kidney Foundation (NKF) guidelines using the last serum creatinine obtained immediately before inclusion in the MASS Registry.

### Treatment protocol

Medications required to achieve adequate values of blood pressure, lipid and glycemic levels, and recommend by guidelines through time were available to patients in all treatment groups. All patients had regular clinical follow-up every 6 months. In this study, all patients were placed on an optimal medical regimen consisting of nitrates, aspirin, beta-blockers, calcium channel blockers, angiotensin-converting enzyme inhibitors, statins, or a combination of these drugs, unless contraindicated. Subjects in the PCI group received bare metal stents (BMS) and first-generation drug eluting stents (DES). Angioplasty was performed according to a standard protocol that included administration of aspirin and a P2Y12 inhibitor agent before the start of the procedure and a minimum duration of dual antiplatelet therapy of 1 month, up to 12 months, according to the type of stent used. In addition, a complete anatomic revascularization was recommended as a main goal.

For patients assigned to CABG, the surgery was performed by select experienced cardiovascular surgeons as the first operators in accordance with the current best practices. The left internal thoracic artery (LIMA) was the first-choice conduit for the left descending artery (LAD) and a complete anatomical revascularization was encouraged. The use of cardiac extracorporeal circulation was defined at the discretion of the surgical team. In those who utilized on-pump surgery, patients had hypothermic arrest and use of cold crystalloid cardioplegia for myocardial protection. In the off-pump surgery, the use of Octopus stabilizer was available.

### Study endpoints

The primary clinical endpoint was the first occurrence of a composite of overall death, myocardial infarction (MI), or additional revascularization (defined as any revascularization, percutaneous or surgical) during follow-up). Secondary endpoints included the total occurrence of each individual component of the primary endpoint.

### Statistical analysis

Quantitative variables were expressed as mean ± standard deviation (SD) or median and interquartile range (IQR) according to their distribution as appropriate. The comparison of means between 2 groups was obtained by using the Student *t* test. When the assumption of normality was rejected, the nonparametric Mann-Whitney test was used. For comparison of means of 3 or more groups, we used the one-way analysis of variance (ANOVA) with multiple comparisons based on the Bonferroni test or the Kruskal-Wallis test, when appropriate. Qualitative variables in each group were expressed as a percentage and compared using the chi-squared test.

Event rates were estimated using the Kaplan-Meier method and differences between renal function strata and therapeutic groups were assessed using the log-rank test.

Multivariate analyses of Cox proportional hazards were used to estimate the risk of the occurrence of outcomes considered in the presence of CKD, as well as the various therapeutic modalities in the different strata of renal function, in an unadjusted as well as adjusted model. In addition, a multivariate analysis was performed to identify independent predictors of the combined primary outcome and overall mortality in the follow-up. In this analysis, variables associated with primary outcome or overall mortality with marginal statistical significance (*P* < .20) in univariate analysis were included in the model. We used the backward stepwise method with criteria of *P* < .05 to remain in the final model.

Interaction analyses evaluating the modification of the effect of treatment modalities by the presence of renal dysfunction on the occurrence of the combined primary outcome and its components were also performed. The statistical significance of differences in the effect of treatment modalities on each endpoint was evaluated using the full population and a multiplicative interaction term.

All tests were two-tailed and *P* values < .05 were considered statistically significant. All analyses were done using SPSS 21.0 software (SPSS Inc, Chicago, IL) for Macintosh.

## RESULTS

The inclusion of patients in the present registry was from October 1995 to May 2010. From an initial number of 6605 patients, 763 diabetic patients with multivessel CAD were selected (Figure 1).

Patients were divided into 2 strata of renal function according to eGFR. Stratum 1 comprised 161 subjects with eGFR values < 60 mL/ min/1.73m^2^. Of these, 42 were in the MT only, 46 in PCI, and 73 in the CABG group. Stratum 2 comprised 602 subjects with eGFR with values ≥ 60 mL/min/1.73 m^2^. Of these, 182 were in the MT only, 158 in PCI, and 262 in the CABG group. The median follow-up time was 9.5 years (Interquartile range 5.5 - 11.2 years). There was no loss of follow-up.

Baseline characteristics were similar among renal function strata, except that patients in the lower eGFR stratum were older (*P* < .001) and less likely to be male (*P* < .001) and smokers (*P* < .001). Comparing the different treatment groups in each stratum of renal function, we observed some differences. In stratum 1, there was a lower proportion of smokers in the PCI group (*P* = .032), and, in stratum 2, there was a lower mean age (P= .015) ang higher LAD proportion (P < .001) in the CABG group, a lower proportion of hypertensive patients in the MT group (*P* = .007), and fewer smokers (*P* = .012), and lower mean HDL value in the PCI group (*P* = 0.010) (Table 1).

**Table 1:** Characteristics of subjects according to treatment group in each stratum of eGFR.

			**Stratum 1 (n=161)** **eGFR < 60 mL/min/1.73m^2^**			**Stratum 2 (n=602)** **eGFR ≥ 60 mL/min/1.73m^2^**				
	**MT (42)**	**PCI (46)**	**CABG (73)**	**Total (161)**	***P* value ^ǁ^**	**MT (182)**	**PCI (158)**	**CABG (262)**	**Total (602)**	***P* value^ǁ^**
eGFR using CKD-EPI (mL/min/1.73m^2^)^*^	48±8	48±9	49±7	48±8	.685	89±13	80±13	81±14	81±13	.073
Male (%) ^†^	61.9	39.1	58.9	54.0	.053	69.8	64.6	70.2	68.6	.441
Age, median (years) (IQR) ^‡^	65 (61-73)	67 (62-72)	65							
(60- 71)	65 (61-72)	.411	63 (55-68)	61 (55-67)	60 (53-66)	61 (54-67)	.015			
Hypertension (%)	78.0	75.0	80.6	78.3	.778	67.3	82.5	73.9	74.2	.007
Smoker (%) ^§^	40.4	17.3	34.2	31.1	.032	48.3	37.3	51.9	47.2	.012
HbA1C, mean ± SD (mg/dL)	8.1±1.7	8.0±								
1.9	7.8± 1.7	7.9±1.7	.540	8.4±7.3	8.0±1.9	7.8±1.9	8.0±4.3	.462		
Glucose, mean ± SD (mg/dL)	161 ± 71	162 ± 54	168 ± 63	165 ± 62	.784	168 ± 63	167 ± 67	160 ± 63	164 ± 64	.420
TC, mean ± SD (mg/dL)	195±51	198±47	201±53	198±51	.834	194±44	191±52	195±47	193±47	.461
LDL, mean ± SD (mg/dL)	117±43	114±41	125±48	120±45	.696	121±39	117±42	121±41	120±41	.406
HDL, mean ± SD	40±13	41±11	39±10	40±11	.615	42±11	39±10	40±11	40±11	.010
TG, mean ± SD	203±156	290± 583	188±69	220±318	.628	173±97	183±107	189±151	182±126	.641
LVEF (%), median (IQR)	64 (60-72)	65 (60-69)	66							
(59-70)	63 (5-69)	.665	64 (55-69)	64 (55-69)	65 (58-70)	63 (55-69)	.147			
LAD (%)	88.1	97.8	97.3	95.2	.101	83.1	86.7	98.1	90.5	< .001
3-vessel, n (%)	78.0	71.7	84.9	79.4	.217	71.6	76.6	79.7	76.5	.147

*: *P* < .001 for comparisons among the renal function strata; ^†^: *P* < .001 for comparisons among the renal function strata; ^‡^: *P* < .001 for comparisons among the renal function strata; ^§^: *P* < .001 for comparisons among the renal function strata; *P* value^ǁ^: for comparisons among treatment groups among each stratum.

CABG, coronary artery bypass graft; CKD, chronic kidney disease; eGFR, estimated glomerular filtration rate; EF, ejection fraction; HbA1C, glycosylated hemoglobin; HDL, high-density cholesterol; LAD, left anterior descending artery; LDL, low-density cholesterol; LVEF, left ventricular ejection fraction; MI, myocardial infarction; MT, medical treatment; TG, triglycerides; TC, total cholesterol.

### Primary and secondary endpoints

**Figure 2:**
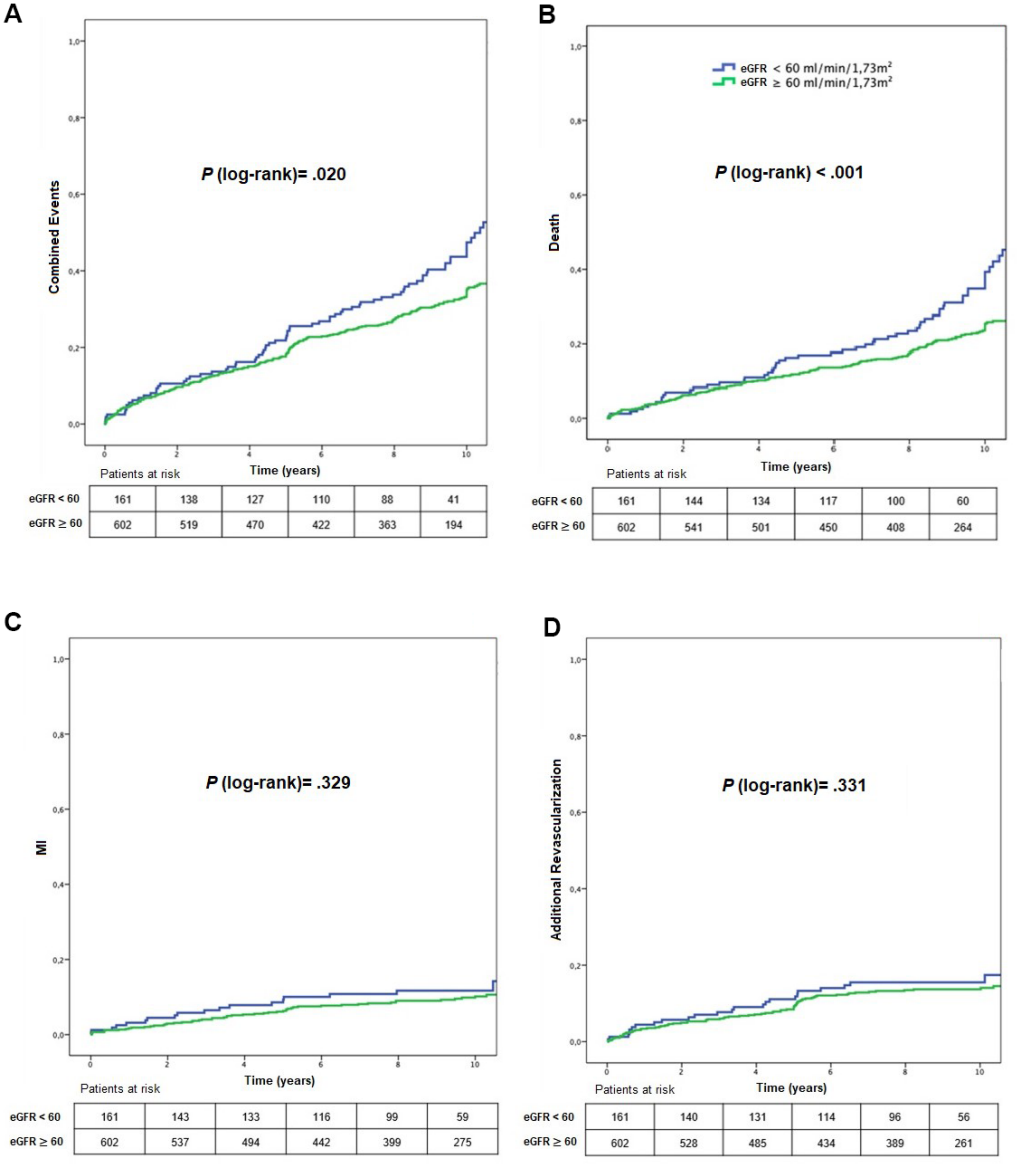
Kaplan-Meier curves showing MACE (**A**), mortality (**B**), myocardial infarction (**C**), and additional revascularization (**D**), according to CKD status. eGFR, estimated glomerular filtration rate; MI, myocardial infarction.

Primary endpoint rates were 53.4% and 40.7% (*P* = .020) for stratum 1 and 2, respectively (HR_adjusted_: 1.51; 95% CI: 1.04-2.21, *P* = 0.030) (Figure 2 and Tables 2, 3). Overall death rates were 37.9% and 24.6% in stratum 1 and 2, respectively (*P* < .001) (HR_adjusted_: 2.10; 95% CI: 1.32-3.35, *P* = .002) (Figure 2 and Tables 2, 3). No difference was observed comparing strata of renal function regarding MI or additional revascularization (Figure 2 and Tables 2, 3).

**Table 2:** Clinical endpoints occurring in 10 years, according to renal function strata and treatment group.

	**Stratum 1 (n=161)** **eGFR < 60 mL/min/1.73m^2^**					**Stratum 2 (n=602)** **eGFR ≥ 60 mL/min/1.73m^2^**				
**Treatment (n)**	**MT (42)**	**PCI (46)**	**CABG (73)**	**Total (161)**	***P* value^‡^**	**MT (182)**	**PCI (158)**	**CABG (262)**	**Total (602)**	***P* value^‡^**
Combined events, n (%) ^*^	32 (76.2)	24(52.2)	30(41.1)	86 (53.4)	.027	99 (54.4)	70 (44.3)	76(29)	245 (40.6)	< .001
Death, n (%) ^†^	21 (50.0)	15 (32.6)	25 (34.2)	61 (37.9)	.410	66 (36.3)	32 (20.3)	50 (19.1)	148 (24.6)	.001
MI, n (%)	7 (16.7)	8 (17.4)	5 (6.8)	20 (12.4)	.070	23 (12.6)	16 (10.1)	25 (9.5)	64 (10.6)	.520
Additional revascularization, n (%)	11(26.2)	13 (28.3)	6 (8.2)	30 (18.6)	.001	46 (25.3)	38 (24.1)	17 (6.5)	101 (16.8)	< .001

*P* values were calculated with the use of log-rank test. ^*^: *P* .020 for comparisons among the renal function strata; ^†^: *P* < .001 for comparisons among the renal function strata; *P* value ‡: for comparisons among treatment groups among each stratum.

CABG, coronary-artery bypass grafting; CKD, chronic kidney disease; eGFR estimated glomerular filtration rate; MI, myocardial infarction; MT, medical treatment; PCI percutaneous coronary intervention.

**Table 3:** Risk of events, death, myocardial infarction, and additional revascularization in different renal function strata.

**Endpoints (Stratum 1 vs 2)**	**Crude HR (CI 95%)**	***P* value**	Adjusted HR (CI 95%)^*^	***P* value**
Combined Events	1.46 (1.14-1.86)	.003	1.51 (1.04-2.21)	.030
Death	1.69 (1.25-2.28)	.001	2.10 (1.32-3.35)	.002
Myocardial Infarction	1.28 (0.77-2.12)	.329	1.16 (0.52-2.60)	.710
Additional Revascularization	1.22 (0.81-1.84)	.331	0.79 (0.41-1.55)	.507

^*^Multivariate models were adjusted for Systemic Arterial Hypertension (SAH), HDL, left ventricular ejection fraction, and glycosylated hemoglobin.

CI, confidence interval; HR, hazard ratio.

### Primary events in stratum 1 and 2 per treatment group

**Figure 3:**
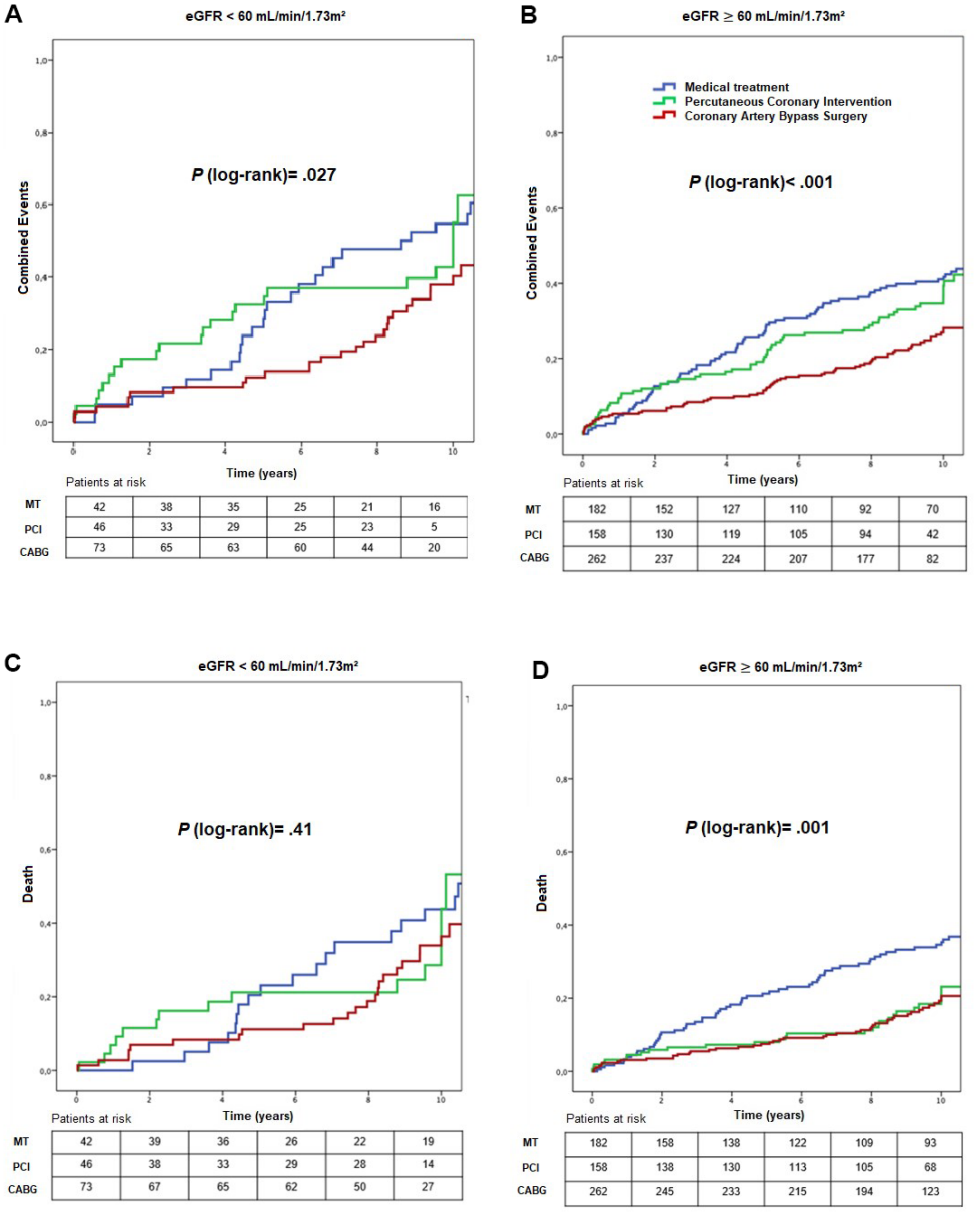
Kaplan-Meier curves showing combined events (**A**, **B**) and death (**C**, **D**), according to CKD status and treatment group. CABG, coronary artery bypass surgery; eGFR, estimated glomerular filtration rate; MT, medical treatment; PCI, percutaneous coronary intervention.

Primary event rates in stratum 1 were 76.2%, 52.2%, and 41.1% for MT, PCI, and CABG, respectively (*P* = 0.027) (Figure 3) (HR_adjusted_: 0.41; 95% CI: 0.18 – 0.95, *P* = .039 for CABG versus MT, HR_adjusted_: 6.51; 95% CI 1.85 – 22.89, *P* = .003 for PCI versus CABG) (Tables 2, 4). Among patients in stratum 2, the primary event rate was 54.4% for MT, 44.3% for PCI, and 29% for CABG (*P* < .001) (HR_adjusted_: 0.59; 95% CI: 0.38 – 0.92, *P* = .022 for CABG versus MT; and HR_adjusted_: 1.96; 95% CI: 1.17-3.26, *P* = .010 for PCI versus CABG) (Tables 2, 4).

**Table 4:** Risk of events, death, myocardial infarction, and additional revascularization in different treatment groups in each stratum of renal function.

		**Stratum 1 (eGFR<60 mL/min/1.73m^2^)**			**Stratum 2 (eGFR≥60 mL/min/1.73m^2^)**		
		**Adjusted HR^a^**	**CI 95%**	***P* value**	Adjusted HR^*^	**CI 95%**	***P* value**
Combined Events	PCI vs MT	2.70	0.82-8.90	.101	1.17	0.71-1.90	.529
	CABG vs MT	0.41	0.18-0.95	.039	0.59	0.38-0.92	.022
	PCI vs CABG	6.51	1.85-22.89	.003	1.96	1.17-3.26	.010
Death	PCI vs MT	0.75	0.14-3.99	.740	0.61	0.30-1.26	.186
	CABG vs MT	0.42	0.15-1.13	.088	0.51	0.28-0.94	.033
	PCI vs CABG	1.79	0.31-10.24	.510	1.18	0.55-2.55	.659
Myocardial Infarction	PCI vs MT	1.54	0.45-5.23	.482	1.20	0.57-2.53	.627
	CABG vs MT	0.35	0.09-1.28	.113	0.99	0.53-1.84	.991
	PCI vs CABG	4.34	1.15-16.21	.029	1.21	0.64-2.39	.578
Additional Revascularization	PCI vs MT	18.58	1.08-319.69	.044	1.89	0.97-3.66	.058
	CABG vs MT	0.22	0.02-1.96	.175	0.39	0.19-0.80	.010
	PCI vs CABG	84.46	3.63-1962.93	.006	4.80	2.28-10.12	<.001

^*^Adjusted for age, sex, smoker status, glycated hemoglobin, LDL, HDL, LVEF, Systemic Arterial Hypertension (SAH) and tri-vessel disease.

CABG; coronary-artery bypass grafting; eGFR, estimated glomerular filtration rate; MI, myocardial infarction; MT, medical treatment;

PCI, percutaneous coronary intervention.

### Comparison of treatments groups in stratum 1

**Figure 4:**
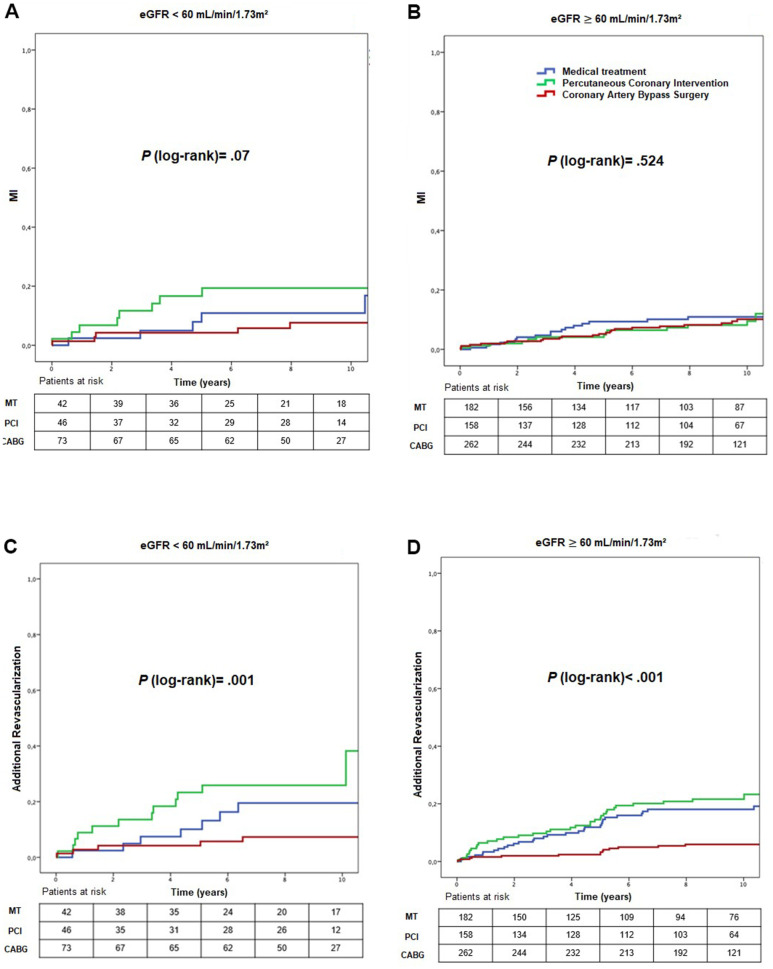
Kaplan-Meier curves showing myocardial infarction (**A**, **B**) and additional revascularization (**C**, **D**), according to CKD status and treatment group. CABG, coronary artery bypass surgery; eGFR, estimated glomerular filtration rate; MI, myocardial infarction; MT, medical treatment; PCI, percutaneous.

Comparing the different treatment, in stratum 1, we found a greater need for additional revascularization in the PCI group (*P* = .001) (Figure 4) (HR_adjusted_: 84.46; 95% CI: 3.63 – 1962.93, *P* = .006 for PCI versus CABG and HR_adjusted_: 18.58; 95% CI 1.08 – 319.69, *P* = .044 for PCI versus MT) (Table 2).

### Comparison of treatments groups in stratum 2

In stratum 2, higher mortality rates occurred in the MT group (36.3%) compared with PCI (20.3%) and CABG (19.1%) groups (*P* = .001) (Figure 3) (HR_adjusted_: 0.51; 95% CI: 0.28 – 0.94, *P* = .033 for CABG versus MT) (Table 4), and a lower need for additional revascularization in CABG compared with MT and PCI groups (*P* < .001) (Figure 4) (HR_adjusted_: 0.39; 95% CI: 0.19 – 0.80, *P* = .010 for CABG versus MT, HR_adjusted_: 4.80; 95% CI: 2.28 – 10.12, *P* < .001 for PCI versus CABG) (Tables 2, 4).

### Interaction analysis

No significant interaction occurred with effect modification on combined events, mortality, myocardial infarction, or additional revascularization, according to the presence of CKD (P_interaction_ = 0.988, P_interaction_ = 0.445, P_interaction_ = 0.369, and P_interaction_ = 0.697, respectively).

### Multivariate regression analysis

A cox proportional multivariate regression analysis was performed and demonstrated that eGFR was an independent predictor of the primary outcome (HR: 0.99; CI 95% 0.98 – 0.99; *P* = .034) and mortality (HR: 0.99; CI 95% 0.98 – 0.99; *P* = 0.020) (Table 5).

**Table 5:** Univariate and multivariate analysis to identify independent predictors of combined events and death.

		**Univariate analysis**			**Multivariate analysis**		
		**HR**	**CI 95%**	***P* value**	**HR**	**CI 95%**	***P* value**
Combined Event	Hypertension	1.43	1.09-1.88	.010	1.52	1.04-2.22	.030
	Smoker	0.91	0.63-1.32	.620	-	-	-
	HbA1c	1.02	1.00-1.03	.056	1.02	1.00-1.04	.038
	eGFR (each 1 mL/min/1.73m^2^)	0.99	0.98-0.99	.006	0.99	0.98-0.99	.034
	LDL (each 1 mg/dL)	1.003	1.00-1.005	.073	-	-	-
	LVEF (each 1%)	0.99	0.98-1.001	.091	-	-	-
	3-vessel disease	0.94	0.73-1.21	.641	-	-	-
	Treatment (PCI vs MT)	0.86	0.66-1.13	.280	-	-	-
	Treatment (CABG vs MT)	0.48	0.37-0.62	<.001	0.54	0.38-0.75	<.001
Death	Hypertension	1.77	1.21-2.57	.003	1.66	1.04-2.64	.031
	Smoker	1.43	1.16-1.77	.001	-	-	-
	HbA1c	1.01	0.98-1.04	.500	-	-	-
	eGFR (each 1 mL/min/1.73m^2^)	0.98	0.97-0.99	<.001	0.99	0.98-0.99	.020
	LDL (each 1 mg/dL)	0.99	0.99-1.003	.730	-	-	-
	LVEF (each 1 %)	0.98	0.98-0.99	.003	0.99	0.98-1.00	.049
	3-vessel disease	1.03	0.74-1.42	.871	-	-	-
	Treatment (PCI vs MT)	0.66	0.46-0.95	.021	0.47	0.26-0.84	.012
	Treatment (CABG vs MT)	0.55	0.41-0.76	<.001	0.56	0.38-0.82	.003

CABG, coronary artery bypass graft; eGFR, estimated glomerular filtration rate; HbA1C, glycosylated hemoglobin; HR, hazard ratio; LDL, low-density cholesterol; LVEF, left ventricular ejection fraction; MT, medical treatment; PCI, percutaneous coronary intervention.

## DISCUSSION

We evaluated diabetics with CAD who underwent different treatments in relation to 2 strata of renal function from the MASS registry. Our results conclude that decreased glomerular filtration rate has a persistent prognostic impact on this population, and that prognostic benefits of CABG were not attenuated in the very long-term follow-up.

This result consolidates - with longer follow-up - current data that have shown that patients with CAD and decreased GFR have worse clinical outcomes compared to groups with better levels of filtration, both in the non-diabetic group and in diabetics in follow-up periods between 3-5 years [9].

As we experience a drop in glomerular filtration, we have a higher level of inflammation and endothelial dysfunction through changes in a series of markers, such as levels of nitric oxide, ADMA, CRP, among others [12]. In addition to these factors, the drop in filtration rate is also associated with non-atherosclerotic cardiovascular risk factors such as myocardial hypertrophy, arrhythmias, electrolyte imbalance, valve calcification and greater propensity for bleeding, which are therefore not fully mitigated by therapy aimed to treat CAD [13]. This combination of risk can explain why we have a had higher mortality rate in comparison to the rates of angina and additional revascularization.

Previous publications comparing treatment strategies in different strata of renal function found no difference in mortality rates among different levels of glomerular filtration levels in a 5-year follow-up [14]. The longer follow-up of this analysis allowed us to observe differences between treatments that did not seem obvious in studies with shorter follow-up.

Differences between treatments became more evident between 4-6 years of follow-up, precisely a critical period that is not evaluated in most studies in this field [9]. The length of the follow-up is extremely relevant because a given treatment can modify its effect over time. Our study shows that surgical therapy was beneficial to this population in both strata of renal function over time. This difference between therapeutic strategies was mainly driven by CABG over PCI and MT regarding combined events in both strata, and regarding death in stratum 2 comparing CABG and MT.

This is in accordance with previous publications that seems to attest to the superiority of surgery in scenarios of patients with more complex anatomy where surgery provides a more complete revascularization, protecting lesions from future obstructions that were initially angiographically insignificant [15].

In addition, the high use of LIMA grafts for LAD in patients undergoing CABG may, in part, explain these results. Although the progression of atherosclerotic disease occurs in the coronary beds of all patients, the impact of CKD appears to be of lesser magnitude on the LIMA, compared with the native beds [16].

Regarding percutaneous treatment, some studies have shown that in this renal population, even with second-generation DES, performs less well than surgical treatment [17, 18]. The greater rate of thrombosis and restenosis in these individuals in the first year as well as progression of atherosclerosis in native beds and neoatherosclerosis may justify a greater rate of events in this population. In addition, antiplatelet therapy also increases the risk of bleeding and can have a negative impact in this population [19].

It is worth mentioning that the benefit of surgery occurred in both strata, which could explain the absence of significant interaction between the kidney function strata and the therapeutic strategies. Baber et al., [17] assessing the impact of CKD among patients in the FREEDOM study, demonstrated that the presence of CKD was associated with higher death rates and adverse cardiac events. However, the presence of CKD did not influence the outcomes found according to the treatment performed. The same result was observed in a collaborative analysis published by Farkouh et al. [18] This meta-analysis demonstrated that surgical treatment had fewer combined events in patients without renal dysfunction and a significant decrease in the need for additional revascularization in CKD subjects in a 5-year follow-up. However, in addition to the shorter follow-up time when compared to our sample, this population also had less anatomical complexity of coronary artery disease, with less than 50% of patients having triple vessel disease, whereas in our sample we have almost 80%.

We found no differences between myocardial infarction and additional revascularization rates between renal function strata. In fact, when we look at the absolute numbers of these events, we notice that there are few events of this type in the follow-up, which could be explained not only by the strict criterion used for myocardial infarction in MASS registry, but by the higher death rates in this subset of CAD population. Besides, additional revascularization was more influenced by therapeutic strategy than by renal function. When we evaluated comparisons between treatment groups, we observed a lower occurrence of additional revascularization favoring patients undergoing surgical revascularization in both groups, which we believe to be attributable to the greater potential of CABG to protect a greater extent of coronary territory when compared to other strategies.

Finally, some considerations should be made. Our study was not a randomized trial, and therefore, there are inherent limitations to this design. In addition, because this was an observational study, we did not have access to the reasons that led to individual treatment strategy indications. Because in our study patients with more advanced CKD, such as eGFR < 30 mL/min/1.73m^2^, renal replacement, or kidney transplant were not included, our results are not applicable for this population.

## CONCLUSIONS

The occurrence of a lower estimated glomerular filtration rate was associated with higher rates of cardiovascular events and mortality, regardless of the therapeutic strategy adopted. CABG was associated with lower combined event rates and additional revascularization at both strata of renal function in a 10-year follow-up.
